# A Primer on Autonomous Aerial Vehicle Design

**DOI:** 10.3390/s151229785

**Published:** 2015-12-02

**Authors:** Hugo H. G. Coppejans, Herman C. Myburgh

**Affiliations:** Department of Electrical, Electronic and Computer Engineering, University of Pretoria, Pretoria 0002, South Africa; hcoppejans@gmail.com

**Keywords:** autonomous, quadcopter, MAV, SLAM, data processing, compression, Microsoft Kinect, stereo cameras, LiDAR

## Abstract

There is a large amount of research currently being done on autonomous micro-aerial vehicles (MAV), such as quadrotor helicopters or quadcopters. The ability to create a working autonomous MAV depends mainly on integrating a simultaneous localization and mapping (SLAM) solution with the rest of the system. This paper provides an introduction for creating an autonomous MAV for enclosed environments, aimed at students and professionals alike. The standard autonomous system and MAV automation are discussed, while we focus on the core concepts of SLAM systems and trajectory planning algorithms. The advantages and disadvantages of using remote processing are evaluated, and recommendations are made regarding the viability of on-board processing. Recommendations are made regarding best practices to serve as a guideline for aspirant MAV designers.

## 1. Introduction

The need for unmanned aerial vehicles (UAVs) in both military and civilian sectors has grown significantly in the last decade [1,2]. The growth in the military sector can be attributed to the recent success that UAVs have had in reducing troop casualties in Iraq and Afghanistan. They play a crucial role in intelligence, surveillance and reconnaissance (ISR) for any large-scale mission. While UAVs are predominantly used for military purposes, civil applications for UAVs are an emerging market. These applications include surveillance, policing, geophysical surveying and transport [3]. When referring to UAVs, a fixed-wing aircraft is typically envisioned, but they also include other types of aircraft designs. Small-scale autonomous aerial vehicles, such as quadcopters, are starting to play a major role in today’s world. The development of these micro-UAVs (MAVs) is becoming popular because of their great agility and ability to perform fast manoeuvres [4].

The most distinguishable characteristic of an autonomous MAV is that it is capable of operating without any human interaction, independent of the environment or mission. An array of sensors is mounted on the MAV and constantly feeds information about the surroundings to the control system that makes intelligent decisions with regard to a specified goal. One of the most important features that an autonomous MAV has is its ability to create 2D and 3D maps of the environment in which it is operating. For the MAV to achieve this, some type of vision system is required. Vision is achieved by a wide variety of sensors that can be mounted on the vehicle. Very expensive sensors, such as laser interferometry detection and ranging (LiDAR) and infrared cameras, are typically used on military MAVs, but in recent years, less expensive sensors, such as the Microsoft Kinect, have been made commercially available and are sufficient for the purpose of returning 3D information of the surroundings. This allows for more flexibility when designing an MAV.

The largest problems that MAVs face are the stabilization and control in six degrees of freedom (DoF), more commonly referred to as attitude and position control. The attitude control can been solved using a simple proportional-derivative (PD) controller. Other techniques can also be applied, such as “sliding mode” and “backstepping” [5,6]. The second problem of position control is much more complex and will be the focus of this paper. Without position control on an MAV, it will be prone to drift because of the constant corrections the attitude controller has to make. The MAV will also be unable to localize itself within a known environment. This problem can easily be solved using GPS [7] and has been the preferred method for UAV designs in the past. For MAVs, however, GPS is not a reliable service, as it is prone to lose accuracy and connection in urban canyons and indoor areas. Because of this, an alternative solution must be found for MAVs.

Some of the earliest methods involve the use of externally-mounted cameras (*i.e.*, off board) to track the movement of the platform. The platform is mounted with reflective tags that the cameras pick up, and by knowing the exact position of the cameras, the platforms position can be inferred [8,9]. This method has numerous disadvantages: the MAV is limited to areas that are covered by the cameras, and the cameras need manual installation and calibration. Thus, this method is limited to areas that are accessible by humans. The only alternative to this method is to use vision sensors that allows the platform to capture the environment surrounding it. This allows the platform to freely explore without a constraint on the area in which it can operate. The difficulty arises when the MAV needs to build a map of its surroundings and then localize itself within that map. A method called simultaneous localization and mapping (SLAM), coined by D. Rye, H. Durrant-Whyte and E. Nebot [10], is the preferred method for solving this problem.

SLAM is the problem of placing a mobile robot in an unknown environment (no prior knowledge) wherein the robot must then be able to incrementally build a consistent map of this environment while simultaneously determining its own location with regard to the map that is being built [11]. The concepts of SLAM have been defined and tested, but the practical realization of these concepts is still an ongoing field of research.

Recently, great strides have been made in creating MAVs that are able to operate in unknown, cluttered and GPS-denied environments using SLAM [12,13,14]. While the problem has been solved to a point where it can be used in a practical application, constant research is being done in order to create alternative solutions or to make improvements. More advanced problems, such as autonomous exploration, swarm navigation and large trajectory planning, are also being researched [14,15,16].

This paper discusses a quadcopter system, tasked with mapping an entire indoor environment. Because the final system consists of so many complex systems that need to be integrated perfectly, this paper presents a complete discussion of all of the core concepts and subsystems and how each subsystem is integrated into the final system. A Microsoft Kinect is the sensor of choice, but alternatives and trade-offs are discussed. This paper focuses on the most recent techniques that are available. Since the choice of hardware is of chief importance, an entire section is dedicated to discussing the hardware requirements.

This paper is structured as follows: The full system design is described in Section 2, while the hardware requirements for the quadcopter platform are discussed in Section 3. The Microsoft Kinect and alternative sensors are evaluated in Section 4, and Section 5 states the advantages and disadvantages of using remote processing. In Section 6, probabilistic SLAM is defined, and a few core SLAM algorithms are discussed. The importance of trajectory planning is discussed in Section 7, and Section 8 aims to provide recommendations regarding the best techniques to use and also to give advice on how to approach the problem of creating an autonomous quadcopter of this kind.

## 2. System Design

Because MAVs are considered to be highly unstable and non-linear systems [13], the choice of sensors, controllers and software is intricate. It is fairly easy to create an ideal model of a quadcopter system, but to design such a system around a set of constraints and specifications is fairly complex. This section presents a theoretical model for the MAV and explains the role of each component.

### 2.1. Basic MAV Structure

**Figure 1:**
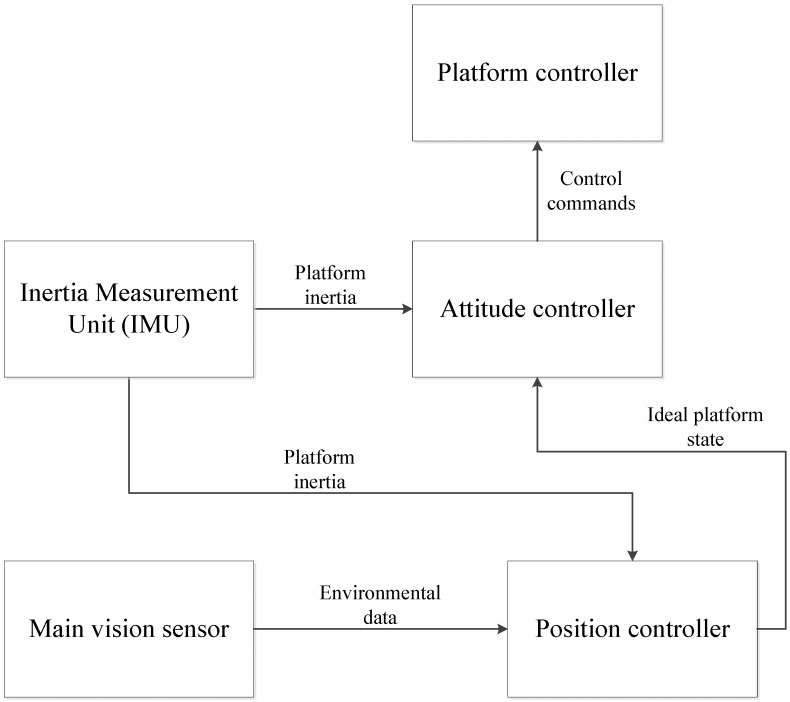
Basic MAV block diagram.

It was briefly mentioned that the most complicated component of n MAV is the attitude and position control. These two controllers are responsible for keeping the MAV in the air and moving towards its goal. Figure 1 shows the interaction of these two controllers with each other and the relevant components.

Attitude control stabilizes the MAV and keeps it in the desired state or position. The attitude controller compensates for environmental factors that cannot be estimated, such as wind and air pockets [17]. It also compensates for small errors in power distribution, caused by non-ideal hardware, which would otherwise lead to the instability of the quadcopter. The attitude controller receives inertia data from the inertia measurement unit (IMU) on the platform, allowing the controller to infer the platform state in free space from the data. The platform’s current state is compared to the ideal platform state that is defined by the position controller. The attitude controller calculates the appropriate changes that need to be made to the platform to achieve the ideal state. While this method is sound in theory, the platform will never be able to achieve the ideal state, but will oscillate around the ideal state instead [17]. The performance of the attitude controller can be quantified by the size of the oscillation.

In a standard human-controlled vehicle, the position controller is replaced by commands given through a controller device. For the purpose of an MAV, the human controller is replaced by a controller system that attempts to reach a goal state. This goal state is either defined beforehand or is changed based on the information gathered during operation. The goal state of the MAV will vary with each new application, so it cannot be explicitly defined. As an example, the goal state of an MAV that is tasked to create a map of the environment will be to move the platform to unknown areas in the map. As each area is scanned, the goal will dynamically change. The position controller calculates the correct power distribution for the motors on the quadcopter platform base so that the platform will start to move towards the goal state at a certain speed. The position controller uses data from both the IMU and the vision sensor to build a map of the environment and to localize the MAV within that map. The data from the IMU are used as a dead-reckoning technique [18], where SLAM is used with the data from the vision sensor. With regard to a UAV or MAV that operates outdoors, the position controller will most likely consist of a GPS. The position controller does not directly send control commands to the MAV, but rather updates the ideal state of the attitude controller.

Typically, the attitude controller needs to run at a much higher frequency than the position controller, because the MAV is so small and agile [19]. A small error in the power output could have drastic consequences, so the MAV needs to stabilize as quickly as possible. The position controller does not need to update as regularly because it is only responsible for moving the MAV from waypoint to waypoint. The position controller also has to run the SLAM algorithms, which are known to be very processor intensive [20]. The attitude controller only has to update based on the raw data obtained from the IMU.

### 2.2. Platform Controller

**Figure 2:**
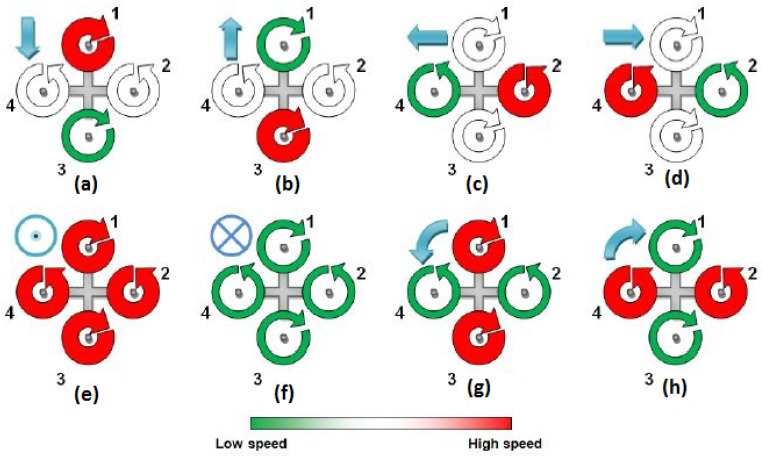
Quadcopter states. (**a**) Forward motion; (**b**) backwards motion; (**c**) movement left; (**d**) movement right; (**e**) increase altitude; (**f**) decrease altitude; (**g**) leftwards rotation; (**h**) rightwards rotation.

The platform controller does not require any type of intelligence or complex algorithms to operate. This controller receives a set of instructions from the attitude controller before adjusting the power distribution between the motors of the platform to perform the received command. Figure 2 shows the possible states that the quadcopter can adopt. Each of these states has a certain outcome, indicated by the blue indicator at the top left of each subfigure. The commands can be hard-coded into the platform controller, and two or more states can even be combined at one time interval. The attitude controller also provides the desired power output for the MAV. The power to each motor is then scaled according to this power output, which allows the MAV to change altitude.

### 2.3. Attitude Controller

As already mentioned, the attitude controller can be implemented using a simple PD controller. This is a very simple and easy to understand process and has proven to be very effective. Normally, the PD controller would be able to run at the maximum frequency (defined by the IMU frequency). Other techniques can also be implemented to solve the problem. One approach is to add a robust compensator to the standard PD controller that allows the controller to restrain the influence of uncertainties. In [21], it is shown that this method is able to bound the attitude controller error to a very small margin.

Two other approaches are “sliding mode” and “backstepping”. A comparison of all three methods was done in [6]. It was found that the PD-controller has a fast stabilization speed, but that the quadcopter converges slowly to a near perfect stable state. The backstepping technique stabilizes even faster than the PD controller, but has a considerably longer processing time, reducing the overall frequency that at which it can run. The sliding mode satisfied the requirements with both the stabilization speed and convergence speed. The sliding mode approach does operate at a higher frequency than the backstepping, but cannot compare to a standard PD controller with regards to updates per second.

The attitude controller only runs on the inertia data obtained from the IMU. The controller can be designed in such a way that it runs on the raw data obtained from the IMU. This would reduce the overall response latency of the system, because it would remove the latency induced by processing the IMU data before using it.

The attitude controller has an “ideal state” that is used to compare with the actual state of the platform. A single state can be represented by the MAV’s speed and acceleration in all six DoF. Six degrees of freedom refers to the MAV’s ability to change its position in 3D space, as well as its roll, pitch and yaw. Thus, the attitude controller receives data from the IMU, which describes the current state of the MAV. This state is compared to the ideal state, and the appropriate commands are generated to change the state of the platform to better match the ideal state. The ideal state is updated by the position controller as the MAV moves around.

### 2.4. Position Controller

**Figure 3:**
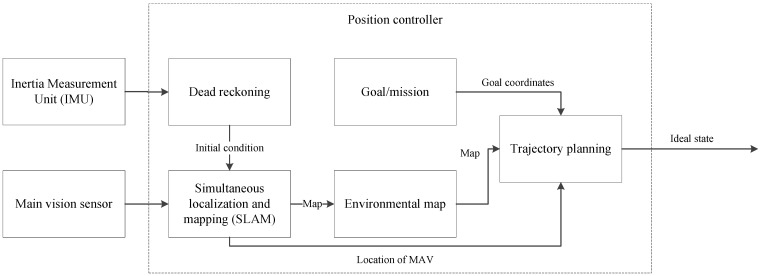
Functional blocks of the position controller.

The position controller is by far the most complex system of the MAV. The role of the position controller has already been briefly discussed, but a much more thorough look is needed. The position controller can be summarized as a controller that takes over the role of a human operator. Thus, it has the very simple task of informing the MAV where to go. For a human, this task is very rudimentary, but to implement it using artificial means is very difficult. Figure 3 shows a breakdown of the position controller into its basic functional units.

The position controller uses data from both the IMU and the vision sensor. The data from the IMU is only used for a single purpose, but it is a very important one. The data obtained from the IMU is used to estimate the movement that the platform has undergone in 3D space between the current instance and the previous one. This method is called dead-reckoning and provides an initial estimate of the MAV’s position. Dead-reckoning is not a very accurate or trustworthy method and on its own does not allow for a very good position estimate, but it allows the SLAM system to be much more accurate. The full extent of the SLAM system will be discussed in Section 6, but it should be noted that if the SLAM algorithms are given an initial estimate of the platforms position, the final output of the SLAM system will be much more accurate and will converge to a solution faster [22].

The SLAM system receives periodical data from the vision sensor of the MAV. The type of data would depend on the sensor that is being used. There is a large variety of different sensors and methods that can be used. The SLAM system must be tailored to work with the type of sensor that is selected. For instance, when a Microsoft Kinect is used, the data would be comprised of a depth image and a red, green and blue (RGB) color image. If a standard stereo camera is used, the type of SLAM system that would be required differs considerable from one using a Kinect [23]. The different possibilities and trade-offs are discussed in Section 4.

The SLAM system returns two pieces of information. With each iteration of the algorithm, a map of the surrounding area is constructed and updated continuously. This map can either be a 2D or 3D map, showing all of the objects and structures that were captured already. This map can be known *a priori*, but the idea of an autonomous MAV is to function in an unknown area; thus, the map needs to be created on the fly. This map will be on scale with the environment around the platform, and the quality will depend on the type of sensor that was used. Because the SLAM system is so time consuming, some implementations purposefully reduce the resolution of the map to increase the frames per second (fps) of the SLAM system [24,25]. The second piece of information that the SLAM system returns is the location of the platform with respect to the map it produced. This allows the system to “know” where it is relative to the objects and structures in its environment. This is a vital part of the MAV’s automation process. This allows the MAV to evade any obstacles and also provides the trajectory planning subunit with an initial position.

The position controller contains some type of goal or mission that it must complete. This goal is very difficult to define, as it would be different for each unique application. One common thread that would be shared between all applications is that the MAV must reach a certain location on the map created by the SLAM system.

The trajectory planner is in itself a complex system that needs to be evaluated. For the purpose of an MAV, the movement of the MAV can be expressed as a holonomic system [26]. A holonomic system is one where the movement of an object can be expressed as a function of position and time such that:

(1) $$f(x_{1},x_{2},x_{3},...,x_{N},t)=0$$

By looking at the MAV as a holonomic system, the trajectory planning can be seen as a kinematic motion planning problem [27], which is discussed more clearly in Section 7. Using this method, the exploration of an unknown environment can be seen as a problem of coverage, where the objective or goal is to visit all of the viable states or frontier states that lie on the boundaries of the map (or as the map is being built, the known free space). Therefore, given an initial state $x_{0}$ of the MAV, the trajectory planning problem is that of finding a sequence of actions that will move the vehicle from its initial state $x_{0}$ to a frontier state $x_{k}$ without any collisions [28]. The biggest problem that the MAV faces is that of knowing when it has arrived at the designated waypoint. When using conventional GPS as a position controller, this problem does not exist, but when working in environments where the platform needs to keep track of its own movements without any external tracking, the problem is a major one. Some of the proposed solutions to this problem propose to work in the belief space in order to distinguish between future states based on the magnitudes of various covariances [29]. This is discussed in detail in Section 7.

Once the trajectory planner has created a set of actions that will allow the MAV to reach its desired location, it updates the ideal state of the attitude controller, after which the MAV starts to execute the actions.

## 3. Quadcopter Platform Base

While the design of the MAV platform might seem to be a trivial part of the overall system, a small error in the construction can lead to the entire MAV failing or at the very least make it impossible to complete the mission. This section defines all of the important hardware aspects that need to be considered. It provides a starting point for any individual that is not accustomed to designing and building MAVs and similar devices. Once again, it should be stated that this particular design is for an MAV that operates in an enclosed environment. The main vision sensor is not discussed in this section, but in Section 4.

**Figure 4:**
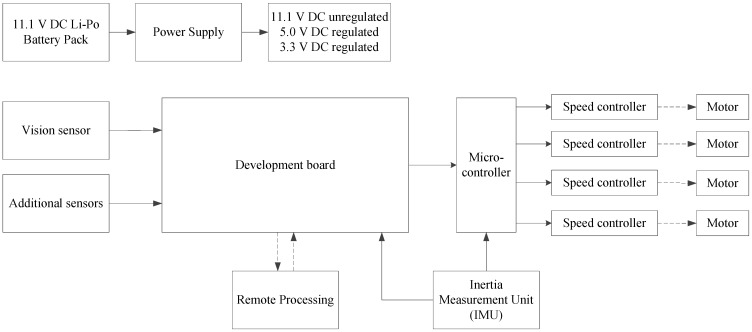
Electrical structure of an MAV.

Figure 4 shows the generic structure of the electrical components for an MAV. The only vital component that is not included in Figure 4 is the frame of the MAV, the only non-electrical component that plays a major role in the overall design. Each component in Figure 4 plays a vital role in the overall design.

### 3.1. Brushless Motors

For the four main motors on the platform, brushless motors are used. Brushless motors are used because they can produce more torque than a brushed motor with the same weight and power usage [30]. The brushless motor is also more reliable and has a longer lifetime. However, the brushless motors use alternating current (AC) power, and thus, they need a more complex and expensive speed controller. All of the brushless motors have what is called a “KV rating,” or voltage constant. The KV rating of a brushless motor is directly related to the theoretical revolutions per minute (RPM) at which it will operate. Equation (2) can be used to calculate the theoretical RPM of a brushless motor:

(2) $$RPM=KV*V_{DC}$$

When constructing an MAV, one of the main design criteria that must be considered is the total weight of the MAV. The motors that are used are directly related to this criteria. The maximum weight of the MAV is determined by the thrust-to-weight ratio that is desired for the MAV. The thrust-to-weight ratio refers to the maximum amount of weight that the motors can hold in suspension. A thrust-to-weight ratio of 2:1 would indicate that the MAV is capable of holding double its own weight stationary. This ratio is determined by the application for which the MAV is being built. Typically, an MAV that will be used for slow, meticulous scanning would need a ratio of 2:1. An MAV that needs to perform high speed manoeuvres in mid air would need a ratio between 4:1 and 8:1. The thrust-to-weight ratio will have a significant effect on the flight time, since the motors would draw much more power for higher ratios.

Another aspect of the motors that should be considered is the available propellers. The propellers, or props, are connected to the brushless motors of the MAV and provide the thrust for take off and flight. Propellers are specified using their diameter and pitch. For all of the props used in multirotor systems, the empirical measurement system is used. The diameter is the length of the prop from tip to tip in inches. The pitch of the prop is defined as the distance that the prop would travel for a single revolution [31]. The larger the pitch of a propeller, the more slanted it becomes and the more power is needed to complete one revolution. A formula for estimating the power that a two-blade propeller uses at a specified RPM is given in [32] as:

(3) $$P=K_{p}*D^{4}*P*RPM^{3}$$

where $K_{p}$ is the propeller constant, *D* is the diameter and *P* is the pitch of the propellers in feet (not inches), and the RPM value is in multiples of 1000. It is good practice to have multiple different types of propellers available while building and testing the MAV. Considering that they are relatively inexpensive, it is useful to have access to blades that can easily alter the amount of thrust provided, as well as having a few spares available.

Each motor would also require an electronic speed controller (ESC) to control the power output to each motor. The ESC will ensure that the motors do not receive too much power and enable each motor to operate independently.

### 3.2. Battery Pack

For MAVs, the most popular battery packs are lithium polymer (Li-Po) batteries. These type of batteries can provide the larger amount of current required by MAVs, while keeping the weight as low as possible. Li-Po batteries also have the property that they can provide a relatively constant direct current (DC) voltage, even when the power level of the battery starts to drop below a level that would be considered as very low [33]. When choosing a battery pack for the MAV, consideration must be given to the desired flight time and the total weight of the MAV. The flight time would be affected by the amount of power that each motor draws (Equation (3)), and the weight will be limited by the constraints put on the motors.

Care should be taken regarding the maximum deliverable current of the battery. The battery should be able to provide enough power for all four motors to run at peak efficiency, or else the system will not be able to meet its designated thrust-to-weight ratio requirement.

### 3.3. Vision Sensor

The vision sensor is responsible for capturing the environment surrounding the MAV. The data obtained from this sensor will be used for the SLAM system. It is imperative that the sensor be mounted in such a way that it can detect obstacles in front of the MAV, or else there will be no way of avoiding collisions. The sensor should be mounted on the bottom of the MAV, so that the frame does not obscure the field of view (FOV).

### 3.4. Development Board

The purpose of the development board is two-fold. It provides the main processing power of the MAV and also connects all of the sensors of the MAV to a central point for processing. The development board is directly connected to the vision sensor, and it periodical receives data that needs to be processed. When constructing MAVs, there are two possibilities with regard to the main processing. The processing can be done on the MAV or the data can be transmitted wirelessly to a remote station for processing. It should be noted again that the SLAM systems used on MAVs are processor intensive, and if the processing is done on the MAV, a capable development board should be used. Development boards that are capable of doing these type of operations are typically much more expensive than their low power counterparts.

If the processing is done remotely, additional latency is added to the system, slowing down the final response time of the MAV. There are also limitation on the wireless transmission throughput, which could result in a bottleneck or loss of data, which will have detrimental effects on the performance of the system.

The development board would be used for compression and data reduction in a remote processing setup. The concept of remote processing *versus* onboard processing is discussed in Section 5. The development board will ultimately be solely or partly responsible for the position controller in Figure 1. The development board also provides an interface between all of the different sensors of the MAV, converting the data into usable information. Ideally, the development board should have a digital signal processor (DSP), allowing for fast image processing on the data received from the vision sensor. Collision detection and trajectory planning would also be implemented on the development board. The development board updates the ideal state of the attitude controller as often as the SLAM cycle and trajectory planning is completed.

### 3.5. Microcontroller

The microprocessor is responsible for the attitude control of the MAV. The data from the IMU should be used directly at the highest frequency that is possible. The microprocessor will most likely function as a PD controller (most popular), comparing the data from the IMU to the ideal state in which the MAV should be. The microprocessor receives state updates from the development board, indicating what the new ideal state should be. The microprocessor then updates the pulse width modulated (PWM) signal that is being sent to the ESCs, changing the power distribution over all of the motors. While the microprocessor does not need to be very powerful, is should however still be able to run the attitude controller algorithms at 10 Hz or higher [34] for a slow-moving MAV.

### 3.6. Inertia Measurement Unit

The IMU is responsible for measuring the positioning of the MAV in a six DoF space. A standard IMU consists of a gyroscope and an accelerometer that return orientation and velocity data, respectively. A standard IMU with only an accelerometer and gyroscope has six degrees of freedom. This would be the three-axis accelerometer readings, as well as the three-axis gyroscope readings. An additional level of accuracy can be obtained by using an IMU that has a three-axis magnetometer, resulting in an IMU that has nine DoF.

There is a large collection of different manufacturers for IMUs, most of which have similar characteristics and specifications. Most IMUs come with a built-in microprocessor that does all of the necessary calculations on the raw data, returning only the relevant data. This removes any additional strain that might be placed on the attitude controller. Alternatively, the attitude controller can work directly with the raw IMU data.

When installing the IMU, consideration must be given to two design criteria that could negatively affect the operation of the MAV. The IMU is very susceptible to vibrations in the airframe, and because the rotors operate at a very high RPM, there will be a significant amount. A simple, but sure way to solve this problem is by insulating the IMU from the airframe with a sponge-like material. The sponge will soak up most of the small vibration, while still allowing the IMU to move with the airframe. The second design pitfall is installing the IMU close to the power distribution board. Because of the nature of an MAV, the power being supplied to the motors fluctuates regularly. This can cause significant interference with the sensors onboard the MAV.

### 3.7. Additional Sensors

While the main vision sensor does provide a large amount of information, most of the sensors available have limitations as to their minimum operational distance, FOV and fps. It is common practice to place 1D or 2D rangefinder devices around the MAV, each one pointing in a different direction. These devices return a straight line distance between the MAV and the closest object, providing distance measurements for landing and collision prevention. This is required because the main sensor might not be able to capture the area directly underneath, above or behind the MAV. These sensors then provide an additional level of safety so that the MAV does not collide with objects and also helps with the take-off and landing procedure.

The most common device that is used for this sensor is an ultrasonic rangefinder. These devices can return the distance to the closest object within a small FOV in front of the sensor. The data from these devices could also be added to the SLAM system, but would not add a large amount of useful information.

One or more additional vision sensors could also be used on the MAV. This would increase the precision that the MAV can operate with, but will make the project much more complex. This would also increase the overall cost of the MAV significantly, since the vision sensor is typically one of the most expensive components.

### 3.8. Frame

The final major component of the MAV is not an electronic device, but the physical frame of the MAV. The frame of the MAV is the component that is influenced the most by the application and specifications. Each available MAV frame differs in some way. The most important specifications of the MAV frame are the weight and size. A frame that is too light would be prone to vibrations, that will interfere with the IMU readings or even break under heavy strain. If the frame is made too heavy, stability will increase, but the flight time of the MAV would be affected or the thrust-to-weight ratio might be too small for flight.

**Figure 5:**
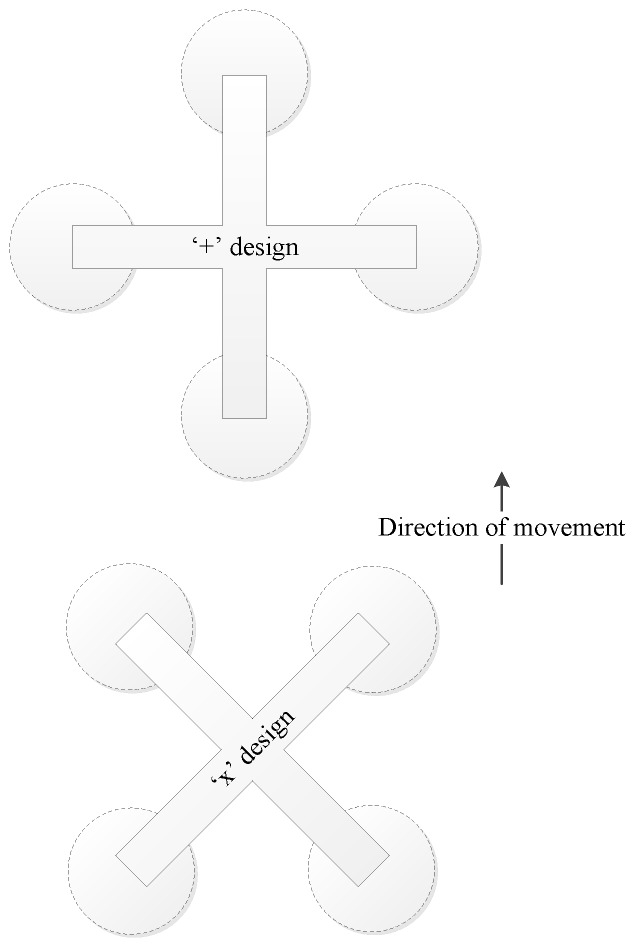
Quadcopter “+” and “x” designs for the same direction of movement.

The frame must also act as a platform for the vision sensor, allowing enough space and a stable mounting bracket. The orientation of the platform must also be considered. For MAVs that are created for indoor use, there must be a size limitation. For indoor use, the biggest size limitation is doorways. By regulation, the minimum width of a doorway is 0.83 m. Thus, the MAV should be able to navigate through a doorway without touching the frame. To account for instability in the MAV, a grace area of more than 0.2 m is advised by [35]. Thus, the maximum diameter of the MAV frame should be 0.63 m. The operational diameter of the MAV can be reduced by using an “x” or cross-orientation, as opposed to the conventional “+” or plus-orientation. Figure 5 shows both of these configuration.

This would make the process of power distribution for movement more complex, but should reduce the operational diameter of the MAV [35].

Before the design is finalized, it must be determined if a gimbal will be attached to the MAV. Gimbals provide a lot more freedom when it comes to scanning the environment, but they tend to add a lot more design constraints to the MAV. A gimbal itself adds a lot of weight to the system, while simultaneously adding the problem of instability. As the gimbal moves the vision sensor, the center of mass for the MAV changes. Because an MAV can easily reposition itself while hovering, the need for a gimbal could possibly be eliminated.

## 4. Platform Vision

The platform vision is the element of the design criteria that will effect the performance of the MAV the most. There exists a direct correlation between the type of vision sensor and the SLAM system to be used. Therefore, careful consideration must be given before the type of sensor is chosen for the platform. There are multiple criteria for choosing one sensor above another. The three most commonly-used sensors are motion sensors for gaming consoles, LiDAR sensors and stereo cameras. The biggest difference between these three devices is the cost. The cost of a stereo camera would be negligible with regard to the other components of an MAV, but using SLAM with stereo cameras is considerably more difficult and does not perform nearly as well as with the other sensors.

Motion sensors for gaming consoles have become more and more popular as of late. With the initial release of the Microsoft Kinect, the research community found that the device could be used for 3D mapping, because it is capable of returning 3D images of the environment. LiDAR devices are at the top of the range sensors, but their cost is significantly more than that of the Kinect or stereo cameras. The accuracy of a LiDAR sensor could however justify the high cost.

The choice of the vision sensor significantly reduces the number of SLAM systems that can be considered. This is because of the major difference in the type of data that are returned by each of the sensors. For each type of data, a different variation of the standard SLAM system must be used. Moreover, each of these variations has alternative implementation methods and algorithms. In Section 6, the most recent SLAM systems for each of the sensors are discussed.

### 4.1. Stereo Camera

**Figure 6:**
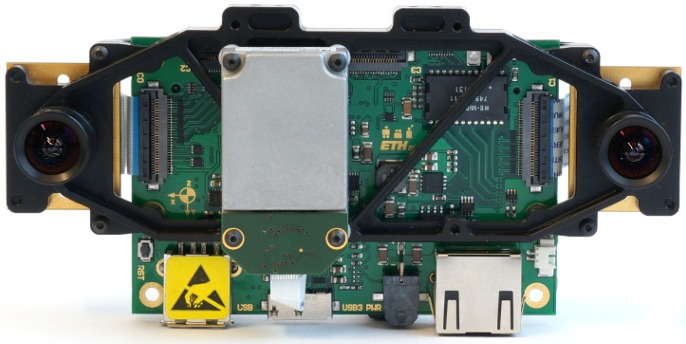
Stereo camera. Skybotix VI-Sensor.

Stereo cameras refer to a type of vision system that captures two images of the same scene, but with a slight position shift between the two images. This attempts to simulate human binocular vision by applying a technique called stereo photography [36]. The exact distance between the two cameras must be known beforehand. The end product is a 3D image of the environment that was captured. The most crucial step in constructing 3D images is the matching of keypoints between the two images. Figure 6 shows a state-of-the-art stereo camera setup designed for UAV navigation.

This method of constructing 3D images does however suffer from large amounts of error. Despite the fact that the field receives a large amount of attention, the methods that are currently being used to correlate two points in the images with each other are both slow and fragile [37]. If this technique is used on a moving platform, the images will also be prone to noise and blur (the noise ratio will depend on the stereo camera) and will introduce ambiguities between matched points [38]. This could make the 3D images unreliable, causing SLAM to fail, leaving the MAV without a clear environmental map.

Another problem one faces when using stereo cameras is illumination. The cameras solely rely on light reflecting off an object. If there is very little light present in the environment (such as a mine shaft or at night), the stereo cameras will fail completely, and the MAV will be rendered useless. Additional illumination can be attached to the MAV, but this would put even more strain on the battery pack and make the MAV very conspicuous. It is proposed in [39] that increasing the exposure time of the cameras could enable the stereo cameras to operate in poor illumination. This would however reduce the frame rate of the system considerably, and the MAV would have to compensate by moving slower.

In comparison with the other two devices, stereo cameras give the worst accuracy and performance, while also adding the criteria of proper illumination to the MAV. The biggest advantage of stereo cameras over the other two devices is the price. The price of two high-quality cameras is neglectable compared to that of the rest of the MAV.

### 4.2. Microsoft Kinect

In recent years, much research has been done on alternative ways to interact with devices. One of the best known devices for this purpose is the Microsoft Kinect for the X-Box. The Kinect is able to track the movement of human limbs in real time using an infrared sensor that captures 3D images of the environment in front of it. The release of this device sparked a huge amount of interest from the research community because of the potential that this device could have in other applications. For our purposes, the Kinect’s ability to capture real-time 3D images of an environment will be used in the SLAM system for the MAV. The Kinect does not only return depth images, but also the corresponding standard RGB images. This allows the MAV to create detailed color 3D images of the environment through which it had traveled.

Since the release of the Kinect, a large amount of work has already been done on this device. Microsoft has released a software development kit (SDK) to support and encourage development using a Kinect. Because the device is readily available and has a large amount of documentation, it is preferred over alternative devices that can perform the same functions.

The Kinect is an affordable sensor that has the capability of producing high-quality images. However, there are a few problems with the Kinect. The Kinect itself is rather heavy and has to be modified before it can be used on an MAV. It also has a physical limitation on the distance at which the sensors can operate. Any object within 0.7 m will not be captured at all and could potentially affect the reliability of the MAV operation. Objects that are further away than 7 m will be prone to a very large signal-to-noise ratio (SNR), which might also cause detrimental effects on the performance of the MAV. The Kinect is also sensitive to sunlight, due to the infrared light from the Sun interfering with the infrared sensor on the Kinect, and will completely distort the depth image that the Kinect returns. Microsoft has produced Kinect for developers, also known as the Kinect for Windows. This version of the Kinect has a near mode that reduces the minimum capturing distance to 0.3 m and provides more functionality to the developer. For the purpose of a budget indoor MAV, the Microsoft Kinect would be the perfect device.

One major disadvantage that the Kinect has compared to a LiDAR sensor is that it has a limited FOV. The Kinect is only able to capture in a small FOV (65${}^{\circ }$). There are multiple solutions to this problem, however. Multiple Kinects can be used, but will increase the final cost of the MAV. The Kinect can also be mounted to a rotating base, allowing it to capture the environment in a complete circle. This would however require the MAV to remain stationary during the rotation. Another, and the most popular solution, is to incorporate this limitation into the SLAM system. The SLAM system is tailored in such a way that it compensates for this limitation and exploits the problem. Because the Kinect is fitted with an infrared sensor, it would be able to work in environments that have no illumination.

The Kinect can return 640×480 pixel depth and RGB images (also referred to as RGB depth (RGBD) images) at 30 fps. This allows the position controller to update the ideal position once every 33 ms. For a slow-moving MAV, this is more than sufficient. This property however poses another problem. Each scan that the Kinect produces is 900 Kb in size. At a full frame rate of 30 fps, the Kinect produces 18 Mb/s, which is a large amount of data for the development board to handle. This aspect of the data is discussed further in Section 5.

**Figure 7:**
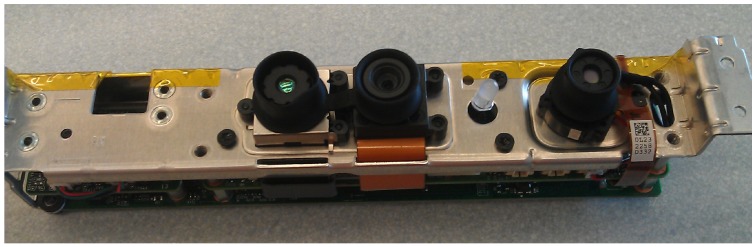
Stripped Microsoft Kinect.

The cost of the Kinect is relatively inexpensive for its functionality. The Kinect might not be able to capture environmental data at long distances, but it is perfect for an indoor environment. The limitations will however prevent the Kinect from operating outside or near any objects that radiate infrared electromagnetic waves. Considering that the Kinect can also be stripped down to a device that weights 115 g, shown in Figure 7, it would not add a lot of unnecessary weight.

Currently, there are alternatives available to the Microsoft Kinect. The ASUS Xtion and PrimeSense Carmine provide much of the same capabilities as the Kinect, but in smaller packages. While these alternatives are much smaller, they do not have the same level of driver quality or data speeds as the Kinect.

### 4.3. LiDAR

The final sensor evaluated in this paper is a LiDAR. LiDAR sensors operate by using laser beams to illuminate a scene and then analyze the reflected light. LiDAR is very popular for UAVs and MAVs that need to create highly-detailed images of large areas. LiDAR is able to provide high accuracy range images over much longer distances than the Kinect. Because the LiDAR operates with lasers on a very unique frequency, it can operate independent of illumination or interference from other sources. LiDAR sensors are also able to scan in a much wider field of view than the Kinect, with some sensors having the ability to create full 360${}^{\circ }$ scans at a high frame rate. The most notable usage of a LiDAR in large-scale scanning is the Google self-driving car. It uses a roof-mounted LiDAR to track every aspect of its surroundings.

LiDAR devices are very complex and difficult to manufacture. For this reason, there are very few firms that produce LiDAR. The cost of a LiDAR device is also significantly higher than that of any of its counterparts. For an MAV that uses a LiDAR sensor, the cost of the sensor will make up the majority of the total cost. The LiDAR devices are also known for their high power usage. Because of this, LiDAR is mostly used on MAVs that are designed for outdoor use and does not have a significant constraint on the size or weight. Most of the lower cost LiDAR sensors (these sensors fall in the same class as the Kinect) only provide range data along a plane around the sensor. Thus, the sensor only detects objects that intersect this plane. These low-cost LiDAR devices therefore struggle in cluttered environments, such as indoor offices.

## 5. Remote Processing

The concept of remote processing has already briefly been discussed in Section 3 and Section 4. When creating an MAV system, there are two possibilities for the main processing. A powerful development board, such as the Pixhawk flight computer, developed by ETH Zurich [40], can be used. This type of development board would typically be a small personal computer, optimized for weight and processing power. The Pixhawk development board provides a 1.86-GHz Core2Duo processor with a total of 4 GB RAM. This type of development board is generally very expensive compared to conventional development boards.

While this method contains all of the processing and components of the MAV, it poses a problem when working with the SLAM system. The SLAM system is very processor intensive and would heavily task the relatively small processor of the Pixhawk. Even in ideal circumstances, the SLAM system will not be able to operate at peak efficiency with such a small processor. While the SLAM system might not struggle, it will not operate as accurately with limited resources. To compensate for this, the SLAM system must be optimized to improve efficiency, which in itself will be very time consuming. However, one advantage that this method provides is that all of the data are centralized on the MAV. This allows the SLAM system to instantly access the data, without any significant transmission latency.

The alternative is to use a remote terminal that does all of the processing for the MAV. While this method eliminates the computational strain from the onboard processor, it does raise its own issues. As an example, the Kinect produces 18 Mb/s of data, and considering that the data must be sent over a wireless link to the remote terminal, it would be impossible to send the raw data using a standard wireless link. Therefore, the received sensor data must be compressed and reduced before it is sent to the remote terminal. The wireless link itself will also introduce a latency to the complete system, which will depend on the wireless connection and how far the MAV is from the remote terminal. The data would need to be received before processing can begin. The MAV would also be limited to an area where there is a wireless signal.

However, this method provides some advantages. The remote terminal would be able to run the SLAM systems much faster, allowing the system to operate at a higher frame rate. The remote terminal can store the full 3D map of the environment and all of the data that were received, while it would be much more difficult to store all of the data when the onboard development board is used. If remote processing is used, it is paramount that the connection between the MAV and remote terminal is both fast and reliable.

### 5.1. Data Compression

For the purpose of a remote terminal, data compression techniques must be discussed. These compression techniques are required because the sensor produces a large amount of data every second, and the data need to be sent over a wireless connection to the remote terminal. The SLAM technique’s accuracy is directly dependent on the quality of the data that it receives, as well as the frame rate of these data [41].

When considering data compression, there are two types of compression, namely lossless and lossy compression. Lossless compression refers to techniques that compress that data in such a way that the original data can be reproduced perfectly from the compressed data, which means that no data are lost during the process [42]. Lossless data compression exploits the repetitive nature of information in the data. Most real-world data contain statistical redundancy where large parts are repeated. These repetitive bit streams are recorded in a codebook, wherein each bit stream is assigned a codeword to represent it. By only sending the codebook once and then encoding the bit stream using the codebook, data compression is achieved [43].

Lossy compression techniques determine which part of the data can be discarded without degrading the quality of the information too much. Each compression technique is solely dependent on its application. For instance, the JPG and MP4 compression schemes are designed for compression of image and video information, respectively. As another example, MP3 is designed for the compression of music. For each of these lossy compression techniques, some “redundant” information is discarded in order to achieve compression, without harming the perception (integrity) of the information [44]. For the purpose of creating an automated MAV, lossy compression should only be considered as a last resort compression technique. This is because SLAM depends on features extracted from the environment, and lossy compression can alter or induce features that do not truly exist.

There are four data compression techniques that should be considered when designing an MAV: DEFLATE [45], bzip2 [42], the Lempel–Ziv–Markov chain algorithm (LZMA) [46] and Lempel–Ziv–Oberhumer (LZO) [47]. These four algorithms provide a wide range of different compression ratios and speeds from which to choose. The choice between these techniques would depend on the particular environment, sensor and the MAV that is being designed.

DEFLATE is a compression technique that uses Huffman coding. DEFLATE provides a good balance between compression ratio and performance. Bzip2 uses the Burrows–Wheeler algorithm to compress data. It can achieve a much higher compression ratio than DEFLATE, but will take longer to compress the data. The LZMA algorithm uses the Lempel–Ziv method of compressing data and falls in between Bzip2 and DEFLATE with regard to compression ratio and compression time. The LZO algorithm provides the least amount of compression, but compresses and decompresses the data much faster than any of the other algorithms.

### 5.2. Data Reduction

If, after compression, the data stream is still too large to send over the wireless link in real time, data reduction techniques must be applied. This would refer to a technique that removes a certain amount of data. There are two simple methods for reducing the amount of data. The first is by reducing the resolution of each image that was captured, and the second is by removing a certain amount of frames each second. This would however have a negative effect on the SLAM algorithm. The accuracy and resolution of the 3D map that SLAM creates will be effected if any of these methods are applied. A method called a voxel grid can also be used to reduce the size of an image. The image is segmented into 3D cubes, called voxels, and in each voxel, only a single pixel may be present. This will remove clusters of 3D point close to each other, evenly spacing most of the points.

Table 1 shows a comparison between various compression techniques using a standard RGB-D image dataset. Microsoft Kinect data were used from the RGB-D SLAM dataset and benchmark [48].

While a higher frame rate is always desirable, it can be noted from experience that beyond a certain point, additional frames add a relatively insignificant improvement to the accuracy of the system. Considering that it is easy to add data reduction to a system, the type of data reduction can be decided on when it becomes clear that there is a need for it. It is quite possible that no data compression will be required, as some SLAM systems can operate on only a few frames each second.

**Table 1:** Compression ratio’s.

Compression Technique	Method	Compression	SD	RGB Size	Depth Size
Original data		100%		900 Kb	600 Kb
Lossless	DEFLATE	41.12%	2.31%	492.6 Kb	124.2 Kb
	bzip2	38.73%	1.75%	471.2 Kb	110.4 Kb
	LZMA	36.4%	1.98%	438.1 Kb	108.4 Kb
	LZO	61.4%	3.47%	675 Kb	246.2 Kb
Lossy	Voxel Grid: 2 mm	43.2%	4.148%	388.8 Kb	259.2 Kb
	Voxel Grid: 10 mm	9.48%	0.78%	85.32 Kb	56.88 Kb

## 6. SLAM

SLAM is the problem of placing a mobile robot in an unknown environment (no prior knowledge) in which the robot must be able to incrementally build a consistent map of this environment, while simultaneously determining its own location with regard to the map that is being built. This problem is one of the cornerstones of creating autonomous vehicles, whether they be land based, aerial or underwater [41]. The theoretical SLAM problem has already been solved, but real-world implementations are almost always much more difficult to solve. The concepts of solving SLAM have been developed and simulated by researchers, but the practical realization of these concepts is still an ongoing field of research.

### 6.1. Probabilistic SLAM

**Figure 8:**
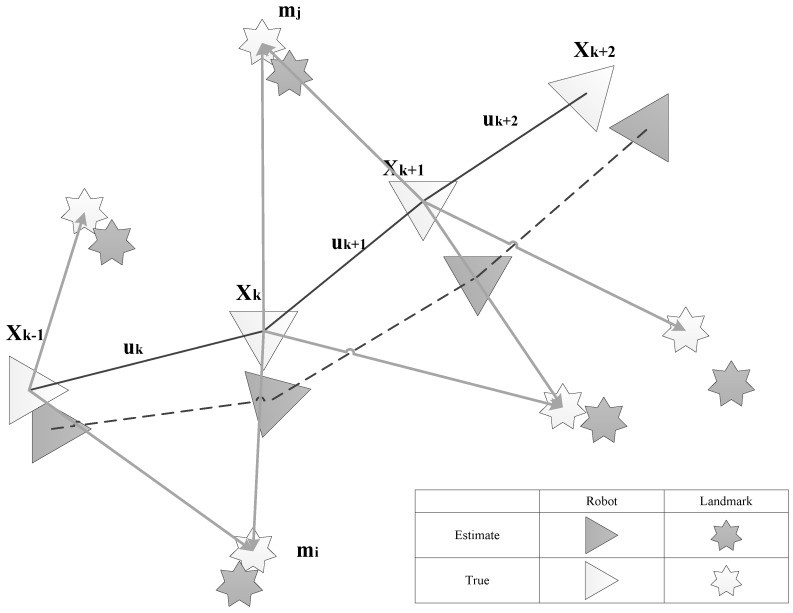
The SLAM problem quantified into different vectors.

SLAM originated by applying estimation theory methods to mapping and localization problems [49]. For a complete discussion of SLAM, the probabilistic model of SLAM should be understood. This suggests that a set of different vectors is defined, each describing a specific characteristic of the SLAM problem. Figure 8 shows the SLAM model used to describe these vectors.

One important fact that must be stated is that the locations of landmarks are never physically measured in the real world and provided to the SLAM problem.

Figure 8 shows a model of the SLAM problem for a robot moving through an environment. For each time instant *k* (typically, this will be for each frame captured), the following vectors are defined.

- $x_{k}$: This is the state vector that describes each location at each time instant. The orientation of the robot is also added to be able to differentiate different FOVs from the same location.
- $u_{k}$: This is the control vector that contains the commands issued to move the robot from the position at time instant k-1 to the current position at time instant *k*.
- $m_{i}$: This is a vector that contains the position of all of the landmarks found in the environment up until time instant *k*.
- $z_{\mathrm{ik}}$: This contains the data from an observation taken of landmark *i* at time instant *k*.

Additionally, we also define the following datasets:

- $X_{0:k}$ = $\left\{x_{0},x_{1},x_{2},....,x_{k}\right\}$: the history of vehicle locations.
- $U_{0:k}$ = $\left\{u_{0},u_{1},u_{2},....,u_{k}\right\}$: the history of command inputs.
- m = $\left\{m_{0},m_{1},m_{2},....,m_{k}\right\}$: all of the landmarks in the known environment.
- $Z_{0:k}$ = $\left\{z_{0},z_{1},z_{2},....,z_{k}\right\}$: the set of all landmark observations.

where Position 0 is to be the initial starting location/landmark.

Using this model of SLAM can be defined as a probabilistic problem that can be solved using Bayes’ equation. For all of the time instants *k*, the probability distribution of:

(4) $$P(x_{k},m|Z_{0:k},U_{0:k},x_{0})$$

must be calculated. This will give the probability distribution that describes the joint posterior density of the landmark locations and vehicle states at the given time instant *k*. The distribution depends on all of the observations and command inputs from the initial position up until time instant *k*.

An iterative process is used to compute the probability distribution in Equation (4) at each time instant *k*, depending on all past time instants k-t, where t=0,1,2...,k-1. The probability distribution can be calculated using Bayes’ equation.

(5) $$P\left(A|B\right)=\frac{P(B|A)P(A)}{P(B)}.$$

Thus, the equation:

(6) $$P(x_{k-1},m|Z_{0:k-1},U_{0:k-1})$$

will give an estimate for the distribution at the time k-1, after a control command $u_{k}$ and observation $z_{k}$ have occurred. Two new models, the state transition model and the observation model, are defined to do this calculation.

The observation model describes the probability that an observation $z_{k}$ is made (at time instant *k*), given the vehicle locations and landmark locations. This probability is defined as:

(7) $$P(z_{k}|x_{k},m)$$

When considering a typical SLAM situation, it can safely be assumed that once the location of the robot and the map is defined, observations are conditionally independent of each other.

The second model that is required is the motion model. The motion model for the robot is defined as:

(8) $$P(x_{k}|x_{k-1},u_{k})$$

It is therefore assumed that the state transitions (or movement) of the robot can be modeled as a Markov process, implying that each state $x_{k}$ is only dependent on the previous state $x_{k-1}$ and the control command $u_{k}$. It is assumed therefore that there are no remaining forces present (such as momentum) on the robot from any states prior to $x_{k-1}$.

Given the observation and motion models, the SLAM algorithm can be implemented in a two-step recursive fashion. The time update and measurement update are done recursively for each time instant. The time update $P(x_{k},m|Z_{0:k-1},U_{0:k},x_{0})$ is given as:

(9) $$\int \left[P\left(x_{k}|x_{k-1},u_{k}\right)P\left(x_{k-1},U_{0:k},x_{0}\right)\right]dx_{k-1}$$

and the measurement update is given as:

(10) $$P\left(x_{k},m|Z_{0:k},U_{0:k},x_{0}\right)=\frac{P\left(z_{k}|x_{k},m\right)P\left(x_{k},m|Z_{0:k-1},U_{0:k},x_{0}\right)}{P(z_{k}|Z_{0:k-1},U_{0:k})}.$$

These two equations give a recursive solution for calculating the joint posterior probability:

(11) $$P(x_{k},m|Z_{0:k},U_{0:k},x_{0})$$

Given the robot states $x_{k}$, the map (or landmarks) m for all of the observations from zero to *k*.

For the next part of the probabilistic SLAM model, the concepts that are presented can be quite complex and confusing. To allow for an easier explanation of the concepts, Equation (4) is reduced to:

(12) $$P(x_{k},m|x_{k}),$$

removing all conditions, except on the locations of the robot. The observation model given in (7) makes it plain that there is a dependency between the observations of the robot location and the landmark locations. Thus, it would follow that we cannot use:

(13) $$P\left(x_{k},m|z_{k}\right)=P\left(x_{k}|z_{k}\right)P\left(m|z_{k}\right)$$

to calculate (12). This fact has been proven multiple times by actual implementation [49,50].

Figure 8 not only shows the actual position of the platform compared to the landmarks, but also the estimated location of the platform together with the landmarks. It can be seen that the error between the actual location and the estimated location propagates from each instance to the next, since the error is in the location of the robot when each of the observations are made. The position of the robot and the position of the landmarks are therefore highly correlated. Because this error propagates with each time instant, the distance between two landmarks or robot positions may be very accurate, even if the actual position is not very accurate. In probabilistic terms, this would suggest that the joint probability distribution of both landmarks $P(m_{i},m_{j})$ has very high peaks, while the marginal probability distributions $P(m_{i})$ and $P(m_{j})$ are more evenly distributed. This was one of the greatest breakthroughs in SLAM research. We can therefore deduce that the correlations between landmark estimates increase monotonically with the size of the observation dataset. For this reason, a map with more sampled data will have greater knowledge about the relative landmark locations.

**Figure 9:**
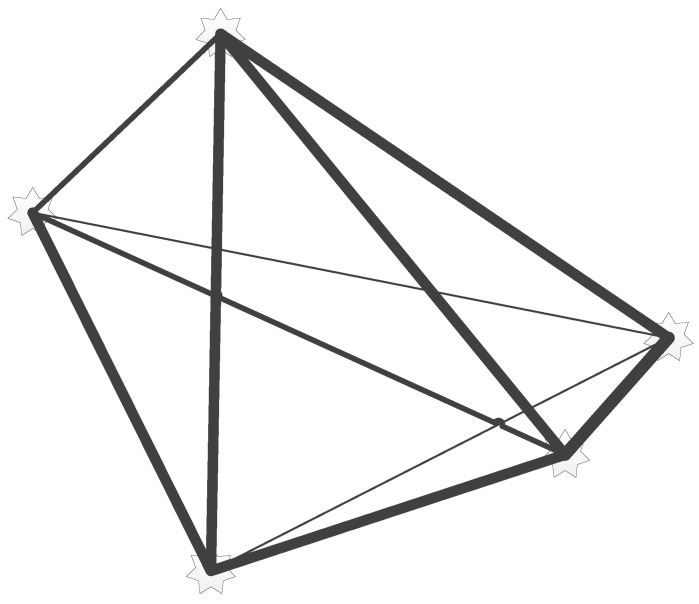
Landmark correlation.

From Figure 8, we can relate a time instant *k* to the robot position at $X_{k}$ and three observer landmarks. When the robot moves to position $X_{k+1}$ during time instant k+1, the robot loses vision of the landmark $m_{i}$. However, since the landmarks are highly correlated, the extra observation of $m_{j}$ will not only increase the estimation of $m_{j}$, but also of $m_{i}$, because of the correlation between the two from previous observations. Thus, every observation of a landmark will effect all other landmark estimations. This links all landmarks as shown in Figure 9.

Although every landmark is correlated to all the of others, the observation of one landmarks might have a more profound effect on some landmarks, while a very small effect on others. This occurs when landmarks are observed during the same observation, thus indicating that they are more dependent on each other, compared to landmarks that are observed separately in different time instances. To illustrate this effect, Figure 9 depicts the effect that the estimation of each landmark has on the others by the width of the connecting line. When comparing Figure 8 and Figure 9, it can be seen that each landmark that is observed during the same time instant has a high correlation, while those that are not observed will have a smaller correlation. The effect will deteriorate as the robot moves further away from a given landmark, thus reducing the correlation.

### 6.2. SLAM Solutions

While the probabilistic SLAM has been defined, it needs to be implemented in real life. This is done by defining an appropriate representation for the observation model and the motion model. These representations must allow for an efficient and consistent computation of the prior and posterior probabilities. There are two basic methods that a large amount of SLAM systems build upon, namely the extended Kalman filter (EKF) method [51] and the Rao-Blackwellised particle filter method [52]. By applying both of these methods to the probabilistic SLAM equations, the EKF-SLAM and FastSLAM techniques are realized. The former method is by far the most popular of the two, but the latter approach, also known as FastSLAM, has received considerable attention in recent years. These two methods provide a basis upon which most state-of-the-art techniques build. While these two methods encompass most of the current-day working systems, there are other methods, such as graph-based SLAM, and hybrids, such as occupancy grid SLAM. While these methods are important in furthering the SLAM research field, it is recommended that newer students start with EKF-SLAM and FastSLAM.

EKF-SLAM was the original solution to the SLAM problem and has been the most widely used, but that does not mean that it is the optimal solution. While EKF-SLAM does perform the best under supervised conditions, it lags behind FastSLAM when it comes to feature-rich (also called landmarks) real-world environments. EKF-SLAM calculations scale exponentially as the number of landmarks increase, while FastSLAM scales logarithmically. EKF-SLAM also lacks the ability to handle uncertainty in posterior probabilities, else the native linearization with extended Kalman filters will induce large errors. FastSLAM, however, poses its own set of problems. The FastSLAM system tends to degenerate with time, thus becoming less accurate the longer it operates [53].

Great care must be given when implementing any type of SLAM system with regards to optimization. Because SLAM operates by correlating landmarks that are similar, the more landmarks you have, the more processing there will be. Thus, any implementation must be optimized with regards to data, as well as programming techniques. Where possible, redundant and unnecessary processes need to be avoided. There are numerous preprocessing steps that can be applied to the data that can reduce the overall redundant data. The amount of time it takes to complete a single iteration of SLAM can greatly affect the final accuracy.

Both EKF-SLAM and FastSLAM are very basic and would not function properly on the MAV without any modifications. The type of modifications that are applied to both of these methods will depend on the vision sensor being used. The general procedure is the same, but there are important aspects that differ from one to the next. There is a very large number of techniques that span over a large number of applications and sensors; thus, focus will only be placed on some of the most relevant implementations.

### 6.3. SLAM on MAVs

Even when using the best performing SLAM method, there will still be challenges running SLAM in real time. This is because the sensors return 3D data, and there will potentially be a large number of landmarks in each scan. With a sensor running at 60 fps, no SLAM system will be able to keep up with the large numbers of frames and key points.

To be able to perform SLAM in real time, a method called localized and partitioned updates is introduced [14]. This implies that the SLAM process is partitioned into localized areas. For short bursts of data capturing (a few seconds), the motion of the MAV is tracked using an iterative closest point (ICP) algorithm [54]. Basic ICP uses brute force to match two successive scans with each other. This method only works when there are minor changes between scans. This allows successive scans from the sensor to be matched to each other from which the relative motion of the platform can be calculated. The SLAM process then only operates on the first image of each block, while using the data obtained from the ICP algorithm as an initial estimate. The initial estimate gives the SLAM system a basic idea of the platforms movement and then refines the estimate. This saves the SLAM system a large amount of computation, while still tracking the movement of the MAV in the frames that are not used for SLAM.

Another major problem that needs to be defined is loop closure. This refers to the ability of a SLAM system to detect when it has reached an area that it has already scanned previously; thus, an MAV that traversed new terrain for a while and has completed a full circle in the environment. The SLAM system must be able to detect the loop and adjust all previous measurements based on this. A SLAM system that detects a loop closure can massively improve the current estimate of its position based on the loop closure and correct for any drift experienced.

Because the type of vision sensor that is used will change the SLAM system, a few different types of SLAM systems are looked at.

### 6.4. SLAM Using Stereo Cameras

Recently, a large amount of research has illustrated that MAVs can operate using standard stereo cameras [12,55,56]. The main purpose of these contributions is to show that an expensive sensor is not needed to create an automated MAV.

The approach followed in [12] is one of the most recent and most successful implementations to date. When using a stereo camera, the localized updates that track the movement of the MAV over a few scans cannot be done using ICP. The local updates and navigation are performed using vector field histogram+ (VFH+) [57]. This method is used because it can deal with large amounts of uncertainty while operating. A secondary rear-facing camera is used for loop closing. The resulting experiments are very encouraging, showing that the MAV was able to do pose estimation, autonomous mapping and exploration. The large-scale SLAM was performed using a remote terminal, since it required additional processing power that only a GPU could provide. It should be emphasized that despite the reported results, there are still a number of challenges, such as detecting landmarks correctly and lighting effects disrupting operation. The tests were performed under controlled conditions; these problems were therefore minimized to show that it is possible to use a stereo camera. Once these challenges have been solved, this method could become one of the best yet.

Another solution, using stereo cameras, was implemented in [58]. This approach made use of a standard EKF-SLAM with the visual odometry (landmarks) and IMU data. While this approach is significantly less advanced than the one in [12], the goal was only to test the MAV’s ability to estimate its own position while hovering. The results showed that the MAV was capable of estimating its own position to within 2 cm. This accuracy can be attributed to the fact that the orientation of the MAV did not change; thus, the landmarks could easily be matched.

**Figure 10:**
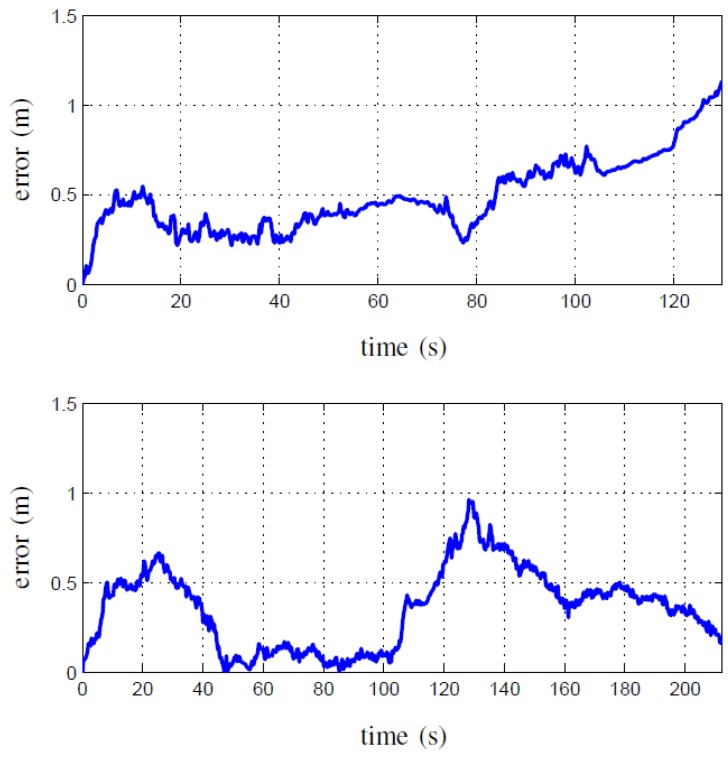
The Euclidean error over time, as measured against DGPS , of a FastSLAM system using SURF features on the first dataset (**top**) and SIFT features on the second dataset (**bottom**) [59].

Figure 10 gives an example of the measured Euclidean error of a stereo camera system behaving in a real-world, noisy environment. A standard robotics platform with stereo vision was used to run experiments with FastSLAM. The Euclidean error was calculated by comparing the estimated position to a Differential GPS(DGPS) module’s data. The results were obtained using both a SIFT and SURF algorithm to detect landmarks. The data given by the DGPS module for ground truth readings are usually accurate to 5 cm. It is apparent from the results that the size of the error is troublesome when compared to the size of an indoor environment. An MAV can easily collide with obstacles with such a large level of error in its position.

### 6.5. SLAM Using a Kinect and LiDAR

Because the data that are generated by the Kinect and LiDAR are very similar, their corresponding SLAM systems are similar. There are only two main differences between the two implementations. The LiDAR returns a gray-scale image that has a much larger field of view, while the Kinect returns colorized (RGB-D) images. The only real difference is they way in which the two techniques calculate the landmarks in the environment. For the Kinect, the landmarks can be extracted using both the depth data, as well as the RGB data. This allows for much more accurate landmark identification. The LiDAR SLAM only identifies landmarks using the depth image, but considering that the depth image has a much bigger FOV, it would be easier to match the current scan to the previous one.

For this implementation, the localized partitioned updates in [14] are once again used. A few papers refer to this method as local and global state estimation [14,60,61]. The local state estimation is responsible for tracking the movement of the platform between each update step for the global SLAM method. The global state estimation is the main SLAM system and does not run as often as the local state estimation. This architecture allows the global SLAM algorithm to use much more processing time than would be possible if the state estimates from the SLAM algorithm were directly used to control the vehicle.

#### 6.5.1. Local State Estimation

For local state estimation, an ICP algorithm could be used. This method has the advantage of being very fast, although it is possible that it might fail due to the speed of the MAV, in which case, the MAV will lose its localized position. An alternative is to use the feature-based approach that is used for SLAM.

**Figure 11:**
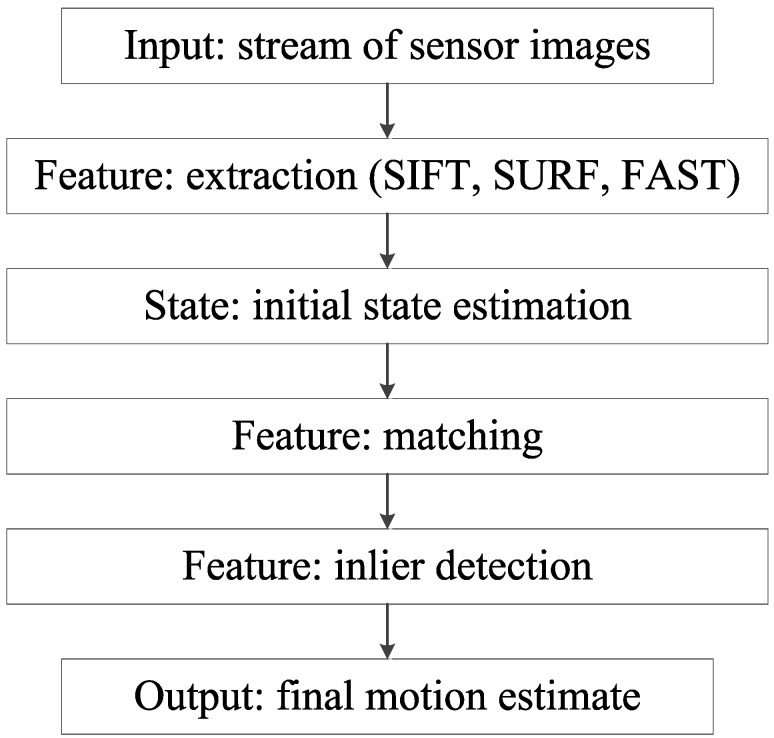
Local state estimation process.

Figure 11 indicates this process. The data that are received from the sensor are used to extract features. Feature extraction refers to extracting landmarks of the environment from the given data. These landmarks can then be used as features for both the local state estimation and the global SLAM.

For feature extraction, three methods are proposed: scale-invariant feature transform (SIFT) [62], speeded-up robust features (SURF) [63] and features from accelerated segment (FAST) [64]. These methods are all based on basic image processing and will not be covered in detail. SIFT and SURF keypoints, while by far the most popular keypoint extraction algorithms for 2D images, rely on local gradients from a unique orientation and do not correspond well over scans from different viewpoints. This is a very important aspect, since you need the ability to detect the same features from different viewpoints to implement loop closure. SIFT keypoints use a combination of color and depth data to detect keypoints, while SURF can either operate with only the color image or both. FAST splits the depth and color image into Gaussian pyramids and then extracts 2D corner features at each level of the pyramid. While [14] suggested the use of FAST features, SIFT and SURF are proven to work with small indoor environments [65].

**Figure 12:**
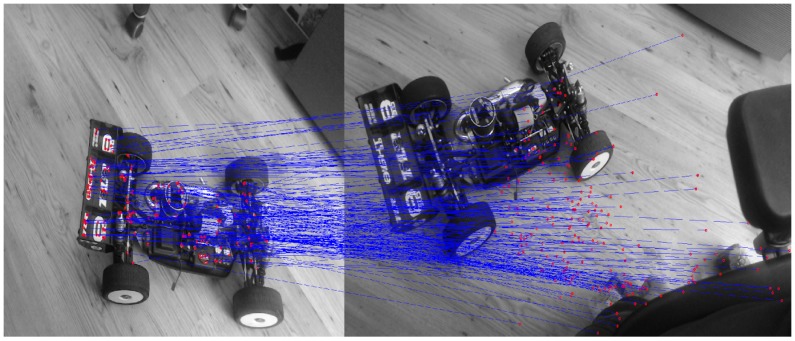
No initial estimate.

**Figure 13:**
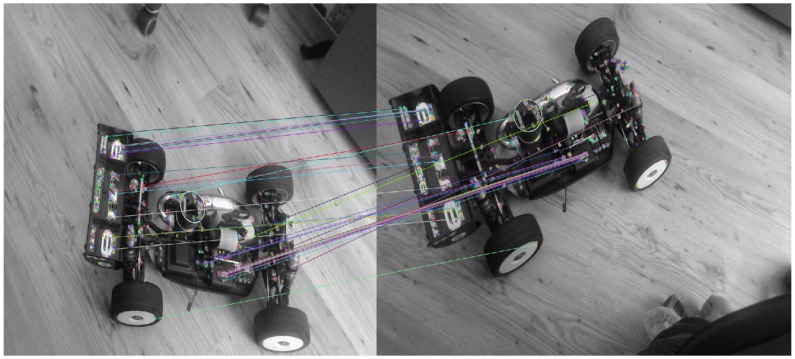
Good initial estimate.

For small motions, such as those encountered in successive image frames, the majority of a feature’s apparent motion in the image plane is caused by 3D rotation of the MAV. Estimating this rotation allows us to constrain the search window when matching features between frames. For our particular platform, the IMU will provide this initial rotation and give us an initial state estimation. Thus, when SLAM tries to match features between two frames, it does not try and match features from one segment of the environment to another separate segment. This is illustrated in Figure 12 and Figure 13. Figure 12 shows feature matching without any initial estimate, where Figure 13 has a good initial estimate, thus limiting the area to which features can be matched. Another solution is proposed in [66] to minimize the sum of the squared pixel errors between the current and previous frame.

For each feature, a descriptor is created that describes what makes that particular keypoint unique. A standard descriptor would be a fast point feature histogram (FPFH) descriptor. FPFH is currently considered one of the best keypoint descriptor algorithms for 3D data. A FPFH descriptor is a representation of the relationship between the keypoint and all of the points surrounding it in a sphere with specified radius [67]. As this sphere around the keypoint increases, the accuracy of the keypoint will increase, but the time to compute it will increase exponentially. Before the FPFH descriptor can be calculated, a surface normal representation must also be computed. Each point must have its normal calculated by looking at the points that surround that specific point and calculating the normal surface across all of them. After each point surrounding the keypoint has a normal estimation, the FPFH descriptor captures the variations in the normals across the sphere around the keypoint.

Although the constraints imposed by the initial rotation estimation substantially reduce the rate of incorrect feature matches between frames, an additional step is necessary to further prune away bad matches. An approach is proposed in [68] where a graph of consistent feature matches is computed, after which a greedy search algorithm is used to approximate the maximal clique in the graph. After the best possible match between features has been found, the final motion estimation can be made. This indicates the translation and rotation that the sensor has undergone between the two frames. The feature matching approach is much more effective and reliable than the ICP algorithm, but it comes with a significant increase in computational complexity, hence an increase in processing time.

The approach depicted by Figure 11 is almost identical to the global SLAM process. However, the number of keypoints that are used for the local state estimation can be reduced considerably, since the movement between two successive scans is much smaller than for the global state estimation. This would also speed up the local state estimation. A combination of ICP and the feature-based approach is used in [69]. For LiDAR implementations, the only major difference is the method that is used to detect features. In [70], a general purpose feature extraction method is proposed that is capable of extracting reliable features from virtually any environment.

One of the biggest problems a newcomer can experience when implementing an autonomous MAV is the feature detection and matching. It is highly advised that this portion of the SLAM system be first implemented separately and fully checked for errors. Any type of problem can make the entire SLAM system malfunction. Extra effort must also be given to optimize the feature matching system as to reduce the number of features that the SLAM algorithms must process. This portion, also known as image processing, can potentially utilize most of the processing power available. It is advised that careful research is done so that the correct feature detection algorithm is used for the data being produced.

#### 6.5.2. Global State Estimation

For the global state estimation, EKF-SLAM and FastSLAM are actually implemented. The global state estimation takes considerably longer than the local state estimation, possible only running once every few seconds. This might pose a problem for MAVs that operate at an increased speed, but for any indoor slow-moving MAV, this method would be acceptable. An alternative to the standard EKF-SLAM is proposed in [71]. An iterated extended Kalman filter (IEKF) is used for the global state estimation. The standard EKF-SLAM is susceptible to errors that are introduced due to the uncertainty of the MAV’s position. For models that are highly non-linear due to measurement, outliers, occlusion and feature errors, the IEKF is able to outperform the standard EKF-SLAM approach.

One major problem that this type of system introduces is that of synchronizing the two-state estimations. Because the local state estimation runs at such a quick tempo, it does not matter if a few frames are missed. On the other hand, it is vital that the global state estimation is never idle. Because the global estimate is reliant on data from the local estimate, the local state estimation must be engineered in such a way that there will always be new data available for the global state estimation.

Once a GPU is utilized to improve the performance of the global state estimate, this problem is exacerbated. The data from the local estimate must already be available to be loaded into the GPU memory before the previous iteration of the global estimate nears its end. If the data are not available, the global estimate process will be stalled, reducing the overall number of frames that can be processed per second.

The number of frames that the entire system can process in real time has a direct correlation to the accuracy of the system. It is difficult to quantify the number of frames for the accuracy, because it varies greatly between implementations. The physical movement speed that the MAV operates on has the same effect that the frame rate has. An MAV that moves too fast will have a much higher error than one that is moving slowly. An ideal MAV platform will operate slowly with a high frame rate. It is possible to increase the velocity of the MAV without losing accuracy by increasing the frame rate proportionally, or if the frame rate is very low, the movement speed of the MAVs can be reduced.

Because there are so many different SLAM systems in use today, a standardized way of comparing SLAM systems using a Kinect is proposed in [48]. A huge online database is provided with Kinect data and ground truth measurements, so that the accuracy of the SLAM system can be calculated. The goal of the dataset was to create a standard database that can be used to effectively test and compare alternative SLAM methods and that also provides data to work with for researchers who do not have access to a Kinect or the correct tools to measure real-world movement.

## 7. Navigation

For any MAV, the ability to move around the environment to reach a desired goal location is called navigation. Navigation is directly reliant on the SLAM system, since the trajectory planning is done on the map created. The navigation must also be done in a 3D coordinate system, since the ability to change altitude will allow the MAV to “climb stairs,” traverse open spaces and pass through openings in a wall. The SLAM system creates a 3D map of the environment that the MAV has traveled through so far. For an MAV that is designated to explore the environment, a frontier-based exploration algorithm is used [28]. This algorithm is fairly old, but has proven to be ideal for these scenarios. Another method that has been derived from the frontier-based exploration algorithm is the probabilistic roadmap (PRM) algorithm [72]. A discrete graph is used to approximate the connectivity of the current position to a possible frontier position. The PRM builds the graph by randomly sampling locations from the map and calculating the connectivity. Multiple points in the discrete graph can be connected to show a path between the current position and a frontier position. Once the PRM graph has found a location to be a frontier position, a collision-free path can be plotted using a graph search algorithm. One problem with the PRM is that the MAV could struggle (due to a bad state estimation) to recognize when it has reached a position in the graph.

One solution proposed by [14] is to work in the belief space, or the space of distribution, where the covariance between future states would indicate the most likely position of the MAV. This method is implemented by using the belief roadmap (BRM) first proposed in [29]. This method proved to be very effective, allowing the MAV to scan an entire office floor without incident.

Another potential problem is the absence of any defining features in the environment. This could make the SLAM algorithm lose track of the MAVs position on the map. Without a clear indication of the position, the trajectory planner would not be able to plot a path. To solve this problem, a wall-following algorithm is used in [12]. This algorithm allows the MAV to keep on following a wall in sparse environments until the SLAM system is able to localize the MAV again.

There will unfortunately be limitation on the size of the area that an autonomous MAV can map. This will partly be because of space limitations and will be determined by the type of hardware used. As the area that has already been scanned increases, the more memory needs to be allocated to that map. The map will be used for both the SLAM system, as well as navigation, so it will constantly be used and change. At some point, the amount of data that needs to be processed will become too large, and the accuracy of the MAV will decline. Currently, much research is being done on how to limit the effects of a large area, but there is no clear-cut solution as of yet.

## 8. Discussion and Recommendations

This paper provided a framework for creating an MAV that is intended for indoor environments. The problem of creating an autonomous aerial vehicle that can navigate enclosed areas has been receiving much attention from the research community in recent years. The large amount of different techniques that could be used to solve the problem can become overwhelming for researchers who are new to this field. This paper strives towards providing a comprehensive discussion on all of the important concepts that need to be considered before an autonomous MAV can be designed.

From all of the facts presented in this paper, it can be said that this is still a very active field of research, particularly with regard to the SLAM systems. None of the SLAM systems that were presented can be said to be perfect. Each one has its own limitations and disadvantages. This is also the case for the type of vision sensor being used. From the literature, it is clear that stereo cameras are by far the least used and the most complex of the three, but have the most potential. If the problems that stereo cameras face are solved, this would provide the least expensive and easiest solution with regard to hardware. It should also be noted that the Microsoft Kinect was originally intended as a gaming device, not as a sensor for development purposes. There are alternative RGBD sensors currently available, but they do not provide any advantage over the Kinect. Ideally, a sensor should be created in the future specifically designed for an inexpensive alternative to LiDAR. LiDAR sensors are by far the best sensors for the creation of an MAV, but unfortunately, the advantages they provide over the Kinect cannot be justified due to the high cost.

Before any design or construction begins on an MAV, a clear set of design criteria should be created with regard to the application of the MAV. A complete implementation (hardware and software) must be planned, so that a clear set of goals can be defined. This will solve a myriad of problems that will be encountered later on in the project if there were no clear design goal in mind. The techniques discussed in this paper are by no means exhaustive, but these techniques can be considered to be some of the best implementations of an autonomous MAV. There might be other, more advanced techniques that might prove more effective in the future.

For any researcher who is new to the field, it is recommended to use the remote processing method. This is because implementing SLAM is already a very difficult process even when little regard is given to the available resources. Implementing SLAM on a system that is very resource limited is considered to be an advanced topic. A very good understanding of SLAM and software optimization techniques is needed. The remote terminal introduces more design criteria and problems to the system, but these problems can easily be solved using a logical approach.

For the attitude controller, there are systems, such as the ArduPilot, that can be bought off the shelf and used in any type of MAV. This allows someone who has only the basic understanding of multirotor mechanics to create an MAV. The system is expensive, but is very powerful. A full IMU is included in the ArduPilot; all that is needed is the physical platform and the main vision sensor. The attitude controller is suitable for any type of environment and is easy to install. The ArduPilot is a system that has been proven to work without fail and would allow a developer to concentrate on systems that are more complex.

## 9. Conclusions

Autonomous MAVs are becoming more prevalent and are already being applied for various applications. The potential of MAVs is near limitless and, over the last few years, has attracted much interest from government and private institutions. There is great potential in the field of MAVs, both for prospecting researchers and veterans. When creating an autonomous MAV, there are many factors that need to be considered. The purpose of the MAV must be defined as multiple design criteria by which the hardware and software must abide. An appropriate SLAM system must be found that allows for operation in the desired environment. The main goal of the MAV must be encoded into the trajectory planner, so that the MAV is capable of completing its objective. While doing all of this, sound engineering logic must be applied. This might seem like an obvious fact, but small errors compound surprisingly quickly into a major design flaw.
